# Sero-prevalence of hepatitis B virus and hepatitis C virus among HIV patients in a suburban University Teaching Hospital in South-East Nigeria

**DOI:** 10.11604/pamj.2013.16.7.3077

**Published:** 2013-09-10

**Authors:** Chiekulie Kevin Diwe, Emmanuel Chidiebere Okwara, Oguamanam Okezie Enwere, Jerome Emeka Azike, Nathan Chibuzo Nwaimo

**Affiliations:** 1 Department of Community Medicine, College of Medicine, Imo State University, Orlu Campus, Imo State, Nigeria; 2 Department of Chemical Pathology, Imo State University Teaching Hospital, Orlu, Imo State, Nigeria; 3 Department of Internal Medicine, College of Medicine, Imo State University, Orlu Campus, Imo State, Nigeria; 4 Department of Surgery, College of Medicine, Imo State University, Orlu Campus, Imo State, Nigeria

**Keywords:** HIV, HBV, HCV, co-infection, seroprevalence, liver disease

## Abstract

**Introduction:**

Highly active antiretroviral therapy (HAART) has improved survival of human immunodeficiency virus (HIV) patients. Concurrent morbidities from liver diseases among these patients have also been observed due to co-infection with hepatitis B and C viruses (HBV and HCV). HAART reduces liver-associated morbidities and mortalities in such patients. Unfortunately free testing of HBV and HCV are not provided alongside free HIV testing and treatment in Nigeria. We assessed the seroprevalence of HBV and HCV among HIV patients presenting for treatment in our center.

**Methods:**

This prospective study of adult patients with HIV/AIDS assessed the seroprevalence of HBV and HCV co-infection using a 19-item questionnaire and collection of 2ml venous blood for hepatitis B surface antigens (HBsAg) and anti-HCV antibodies. All previously diagnosed HIV patients of the unit were excluded from the study.

**Results:**

Of the 404 patients, 69.2% were females while 30.8% were males. Married participants were 59.6%, 25.3% were single and 15% were previously married. A large proportion (69%) of patients were farmers, artisans and traders. Most had some formal education; secondary (55.3%), primary 27.3%, tertiary 13.8%. HBsAg positive participants were 9 (2.2%) while 3 (0.7%) were positive for HCV. No participant had triple infection of HIV/HBV/HCV.

**Conclusion:**

Seroprevalence of HBV and HCV is low among HIV patients in Orlu. However there is a need for HBV and HCV testing of all HIV positive patients to reduce morbidities and mortalities from liver diseases.

## Introduction

The introduction of highly active antiretroviral therapy (HAART) has improved the survival of human immunodeficiency virus (HIV) infected patients. Concurrently, mortalities primarily from liver diseases among these patients now top mortalities from non-AIDS causes [1]. The increase in mortalities and morbidities from liver diseases amongst HIV patients is in part due to co-infection with hepatitis B and C viruses (HBV and HCV) as these viruses promote liver fibrosis by increasing intra-hepatic apoptosis [2, 3]. The risks associated with HIV transmission are similar to that of HBV and HCV. The AIDS virus depletion of gastrointestinal tract associated CD4 lymphocytes also make the gastrointestinal mucosa to become more permeable thereby causing microbial translocation and liberation of endotoxins such as lipopolysaccharide (LPS) which is pro-inflammatory and pro-fibrotic on the liver [4, 5]. It is also well known that liver toxicity from HAART exist but does not override the overall usefulness of HAART. With the HAART-associated reduction in deaths primarily due to AIDS, the emphasis now is on reducing deaths primarily from liver diseases among HIV patients. One of such strategy is screening of all HIV patients for HBV and HCV. In a study 49% of HIV patients with abnormal liver function test have well identified liver disease [6]. Clearance of HCV was found to be less likely in HIV infected subjects as well as less likely in Negro populations [7]. The World Health Organization (WHO) recommends that HAART should be commenced in HIV patients co-infected with HBV or HCV irrespective of value of CD4 count [8]. This reduces liver-associated morbidities and mortalities in such patients. Of 86 HIV positive HAART naïve patients who were followed up for 6-36 months in a study in Naples, 32.5% had hepatic flares out of which 64.7% of them where positive to HBV DNA compared with 24.6% who were negative [9]. Unfortunately, in resource poor countries such as Nigeria, free testing of HBV and HCV are not provided alongside free HIV testing and treatment in many centers. This is the case with our center. The implication is that HIV patients whose CD4 counts are above 350cells/ml but who may be positive to HBV or HCV are unrecognized and do not have early commencement of HAART with damaging consequences on their liver. We assessed the seroprevalence of HBV and HCV among HIV patients presenting for treatment in Imo State University Teaching Hospital (IMSUTH) Orlu with a view to identifying the proportion of such patients co-infected with these hepatotrophic viruses. This work is also intended to add to the literature of seroprevalence of HBV and HCV among HIV patients in our locality.

## Methods

This study was a prospective study conducted at the Imo State University Teaching Hospital, Orlu, Imo State, South-Eastern Nigeria, a tertiary care hospital located thirty (30) kilometers from the state capital, Owerri. Orlu is a semi-urban town with an estimated population of 220,000 [10]. The hospital is a 200-bed teaching hospital serving as a training center for undergraduate medical students, resident doctors and nursing students. The hospital has a unit in the Community Medicine Department dedicated solely to free voluntary testing of HIV, free counseling and free drug treatment of HIV infected individuals and treatment of tuberculosis. The study population was confirmed HIV positive adult patients who presented newly at the HIV treatment unit of the hospital from May 2012 to July 2012 (3 months period). Only those who gave informed written consent to participate in the study were recruited. A 19-item structured self-administered questionnaire was given to all participants. Questionnaires were interviewer-administered to participants who had difficulty in responding to the items. The identity of participants was protected by allocating identity numbers to them and participants’ names were not required. Questionnaires obtained basic demographic data, previous history and risks associated with transmission of HIV, HBV and HCV infection. Each completed questionnaire was retrieved immediately by the study investigators. Thereafter, 2ml of venous blood was collected from each participant in a sterile plain tube. After clotting and retraction samples were centrifuged at 250 revolutions per minute and the serum was separated using Pasteur's pipette and tested for HBsAg and anti-HCV antibodies. Specimens which could not be tested immediately were stored frozen at -8oC in the refrigerator until laboratory analysis the following day. Testing of each participant's serum for HBsAg and HCV antibodies were done using ACON laboratories (San Diego, California, USA) rapid screening kits, following the manufacturer's instruction. Each test kit is a lateral flow qualitative immunochromatographic assay.

Four hundred and four (404) consecutively recruited adult patients participated in the study. The data collected was entered into a password protected computer and analyzed using SPSS version 16. In the statistical analysis, frequencies, mean values and percentages were presented.

## Results

There were 404 HIV positive patients who participated in the study with a mean age of 38.3years (±9.8). Of the 404 participants, 69.2% were females while 30.8% were males. Married participants comprised 59.6%, 25.3% were single and 15% were previously married. Commercial drivers constituted 7.6%, civil or public servants 12.9%, students 10.5% while 69% were any of subsistence farmers, artisans or traders. Highest formal education attained was secondary (55.3%), primary 27.3%, tertiary 13.8% while 3.6% had no formal education. Christians were 95.9%; 2.9% were Muslims and 1.2% were had no religious inclination. HBsAg positive participants were 2.2% (9/404) of all participants (3.2% of all males and 1.8% of all females) while 0.7% (3/404) was positive for HCV (all were females). No participant had triple infection of HIV/HBV/HCV.

## Discussion

In 2008, in Georgia, almost half (48.57%) of HIV positive patients were found to be co-infected with HCV while 43.42% were co-infected with HCV and 5.14% had triple infection of HIV/HBV/HCV [11]. Men were more likely than women to be co-infected but infection was associated with sharing of injection equipments amongst drug addicts. This prevalence values are undoubtedly very high. In Slovenia, in the same year 10.7% of HIV positive patients were co-infected with HCV while 25.5% were co-infected with HBV and 0.56% had triple infection [12]. These values are comparably lower than the values observed in Georgia. In the same year in New York City, 25% of HIV patients were co-infected with HCV while 4.47% were co-infected with HBV and about 1.6% had triple infection [13]. In 2005 in Australia prevalence of HCV co-infection amongst HIV patients was 12.8% while HBV co-infection was 4.8% [14]. The Australian values are lower than the rest. We reported HCV co-infection prevalence of 0.7%, HBV co-infection of 2.2% and no triple infection in this study. This perhaps may be related to the low incidence of intravenous drug abuse and needle sharing amongst our people compared to the developed countries. Our study was done in a suburban environment with 69% of study population being either subsistence farmers, artisans or traders. These attributes of our study population may account for the lower prevalence values we observed. Also, in most of the studies, the prevalence of HCV co-infection was higher than prevalence of HBV co-infection while the reverse was what we observed. In India, in 3 different studies, HCV co-infection / HBV co-infection amongst HIV patients were respectively 2.1% / 6.4%, 2.2% / 9% and 1.69% / 2.61% [15–17]. These values are much lower than the European values stated above but closer and yet lower than the observation in Australia. However, these Indian prevalence values although higher than the values we reported in our study show a trend of HBV co-infection being higher than HCV co-infection and perhaps could be a characteristic finding in developing countries.

In a study of 378 HIV positive individuals in Nairobi, Kenya 6% were co-infected with HBV while 1% was co-infected with HCV [18]. While in a cohort of 138 HIV positive patients in Ghana HBV and HCV co-infection respectively were 13% and 3.6% [19] and another cohort of 126 HIV positive individuals in Mekelle, Ethiopia 9.2% were co-infected with HCV [20]. These prevalence values are higher than our observation. In a meta-analysis of studies reporting HBV and HCV prevalence data amongst HIV patients, sub-Saharan African mean prevalence was reported as HBV co-infection 15% while HCV co-infection 7% [21]. That study concluded that HIV is associated with a higher prevalence of both HBV and HCV but this association is less evident than that observed in Western countries.

In Jos, Nigeria among a cohort of 258 clergymen-in-training 2.7% were HIV positive of which 0.4% had HBV/HIV co-infection, none had HCV/HIV co-infection and 0.4% had HCV/HBV co-infection [22]. These subjects represent a classical low risk group and this could account for the very low prevalence observed when compared to our study despite the urban environment of Jos, Nigeria. In a cohort of 260 HIV positive individuals in Abuja, Central Nigeria 11.5% were co-infected with HBV, 2.3% were co-infected with HCV while 1.5% had triple infection [23]. The cosmopolitan nature of Abuja as well as cultural differences as it concerns marriages may account for this. Whereas among 300 male prisoners in Nasarawa, Nigeria 2.7% had HIV and HBV co-infection while 0.7% had HIV and HCV co-infection [24] values comparable to as we observed despite the difference between the two study groups.

## Conclusion

A low seroprevalence of HBV and HCV is associated with HIV infection in Orlu, suburban south-eastern Nigeria. Therefore HBV and HCV testing should be included in the protocol of all HIV treatment centers even in resource limited settings in order to reduce morbidities and mortalities from liver diseases amongst HIV positive patients.

**Limitations:** HBV DNA and HCV RNA by polymerase chain reaction were not done due to unavailability of required technology. This may have increased the prevalence of HBV and HCV in our study as it would allow early diagnosis of these infections before surface antigen of HBV or anti-HCV antibodies were detectable in serum.
